# Exploring the roles of academic expectation stress, adaptive coping, and academic resilience on perceived English proficiency

**DOI:** 10.1186/s40359-024-01630-y

**Published:** 2024-03-16

**Authors:** Po-Chi Kao

**Affiliations:** Center for General Education, Chang Gung University, Taoyuan City, Taiwan

**Keywords:** English proficiency, Academic expectation stress, Adaptive coping, Academic resilience, EFL education

## Abstract

This study aims to examine and analyze a research model comprising three latent variables (academic expectation stress, adaptive coping, and academic resilience) to gain insights into the perceived English proficiency of EFL (English as a foreign language) learners. These variables have been overlooked in previous literature despite their importance in understanding learning outcomes. A total of 395 undergraduate students from a Taiwanese university participated in this study. Through the use of structural equation modeling, the hypotheses in the research model were tested. The findings of this research are as follows: (1) Academic expectation stress has a significant and negative impact on EFL learners’ perceived English proficiency; (2) Academic resilience positively predicts EFL learners’ perceived English proficiency; (3) Academic resilience mediates the relationship between academic expectation stress and perceived English proficiency; (4) Adaptive coping mediates the relationship between academic expectation stress and academic resilience. These results add valuable insights to the existing literature in EFL teaching and learning, shedding light on the dynamics of these variables.

## Introduction

The quest of English proficiency for EFL (English as a foreign language) learners can be a difficult road fraught with challenges. Academic expectation stress and academic resilience stand out as elements that are likely to influence students’ learning performance among these obstacles [1, 2]. Adaptive coping may also play a role [3]. Academic expectation stress is defined as the psychological strain experienced by students due to the demanding nature of academic expectations imposed upon them [6]. Academic resilience is defined as the capacity of students to successfully deal with difficulties, setbacks, and stressors while retaining a positive outlook, adaptability and perseverance [9]. Adaptive coping is defined as the proactive approach of actively taking actions to eliminate or overcome stressors and reduce their impact [3]. The purpose of this study is to investigate and analyze the complex relationship between academic expectation stress, adaptive coping, academic resilience, and perceived EFL proficiency. The author hopes that this study will shed insight on the underlying mechanisms of latent variables that may affect students’ EFL learning outcomes directly or indirectly.

As the importance of mastering English in today’s society is widely acknowledged, the interplay of psychological factors and language acquisition is becoming an important research area in the study of psychology and language education. While previous researchers have made attempts to investigate the dynamics of psychological factors and language learning, there are still novel factors to explore, particularly to test if these factors can contribute to or impede the development of English proficiency. By delving into the exploration of these latent variables, this study seeks to provide a more comprehensive understanding of the multifaceted nature of language learning.

Moreover, this study goes beyond a unidimensional examination of individual factors and instead explores the synergistic dynamics of these factors. By doing so, this study offers a richer perspective on the mechanisms shaping EFL learning outcomes, thereby advancing the current understanding in this field. By understanding the issues faced by EFL learners in their quest for English proficiency, this study can ultimately contribute to the development of more effective pedagogical approaches in language education.

## Literature review

In light of the significance of enhancing English proficiency among EFL learners, this study reviewed previous research and theoretical frameworks, aiming to examine the above-mentioned underlying variables that may shape and influence perceived English proficiency among EFL learners. Through the review and discussion of relevant literature in the following sections, the author aspired to illuminate the complex pathways that may underlie EFL learners’ journey towards English mastery.

### English proficiency

In today’s globalized world, English ability has become a vital asset. It provides individuals with educational, professional, and business opportunities, allowing them to succeed in an interconnected society. Mastery of the English language allows people to converse effectively with others from various backgrounds and improves intercultural understanding. English proficiency has become a precondition for success and upward mobility as it continues to dominate professions such as science, technology, commerce, and academia [4]. By understanding the importance of English competence and the elements that influence it, EFL learners can realize their full potential brought by mastering the English language.

In light of the significance of enhancing English proficiency among EFL learners, this research project aims to investigate the impact of underlying variables that have received limited attention in prior language education studies. Additionally, this study responds to the call made by Gardner [5] to explore novel variables in research on foreign language teaching and learning. Building upon previous research, this study introduces a conceptual framework comprising three factors that could have a direct or indirect influence on the perceived English proficiency of EFL learners. These latent factors include academic expectation stress, adaptive coping, and academic resilience.

### Academic expectation stress

Academic expectation stress refers to the psychological strain experienced by students due to the demanding nature of academic expectations imposed upon them [6]. Teachers, parents, classmates, or even self-imposed pressures can all contribute to these expectations [7]. Fear of failure, the quest of high grades, competition, and the need to satisfy social or personal success criteria are all common causes of stress [4]. These expectations can be overwhelming, resulting in anxiety, self-doubt, and a lower feeling of well-being [6].

Academic expectation stress can have a substantial impact on students’ learning experiences. Excessive or chronic stress inhibits cognitive functioning, impairs attention and concentration, and disturbs memory processes [8], all of which are necessary for EFL learning. Students may also suffer diminished motivation, lower interest in learning activities, and a drop in academic achievement [8]. As a result, its negative consequences on academic performance might create a downward spiral, exacerbating EFL learners’ English learning challenges.

### Adaptive coping

Students may use a variety of coping mechanisms to overcome stressful situations when faced with academic expectation stress. Adaptive coping is one such strategy. It refers to the proactive approach of actively taking actions to eliminate or overcome stressors and reduce their impact [3]. Active and planning tactics are used in adaptive coping [3]. The active method entails taking the initiative to tackle challenges, make efforts, and systematically implement coping techniques [3]. The cognitive process of considering how to successfully deal with a stressor is referred to as planning. It comprises devising action-oriented plans, deliberating on the essential measures to solve the issue, and deciding the best strategy to deal with the situation [3]. Adaptive coping represents a coping approach that places great importance on assuming control and actively tackling stressors. It entails acknowledging the presence of stress, comprehending its nature and consequences, and purposefully taking measures to mitigate and conquer it [3]. The strategies employed in adaptive coping concentrate on effectively addressing the underlying causes of stress, while simultaneously cultivating the necessary skills to triumph over challenges. This method fosters a sense of self-assurance, fortitude, and personal empowerment when confronted with adversity [3].

### Academic resilience

Academic resilience is the capacity of students to successfully deal with difficulties, setbacks, and stressors while retaining a positive outlook, adaptability and perseverance [9]. Self-belief, self-control, optimism, and a growth mindset are among the psychological qualities possessed by resilient students [10]. Academic resilience can be nurtured and developed through supportive environments, healthy relationships, and targeted interventions [9].

Students who are resilient are more inclined to uphold a balanced outlook, establish objectives that are attainable, and tackle obstacles with a proactive mindset for finding solutions [11]. According to Martin & Marsh [12], academic resilience enables students to recover from setbacks and embrace efficient study techniques, resulting in enhanced scholastic achievements and a more gratifying learning journey.

## Rationale for the study

The literature review exposes a research gap in terms of limited attention to certain variables in the context of language education. Prior literature elucidates the detrimental effects of academic expectation stress on students’ learning experiences and academic achievement. Excessive stress can disrupt cognitive functioning, impair attention, and disturb memory process, which in turn may adversely affect EFL learning. Understanding the impact of academic expectation stress on language learners is crucial for effective language instruction. In addition, previous literature also highlights the significance of adaptive coping and academic resilience. Adaptive coping strategies, as mentioned, are crucial for addressing stressors and assuming control. Academic resilience, with its emphasis on a positive outlook, adaptability, and perseverance, plays a pivotal role in students’ ability to recover from setbacks and enhance their learning performance. While existing research has laid a strong foundation for understanding these factors respectively, there’s a need to delve deeper into the associations between these factors and English proficiency. The associations between these variables have received limited attention in prior research on language education. This research intends to bridge this gap by investigating these variables and their potential influence on English proficiency. In particular, the present study aligns with the call made by Gardner [5] to explore novel variables in research on foreign language teaching and learning. By introducing a conceptual framework that incorporates these understudied factors, the research responds to the academic community’s call for a deeper understanding of the multifaceted nature of language learning. The research also seeks to address the identified research gap and provide a more comprehensive understanding of the dynamics involved in EFL learning. This knowledge will not only contribute to the field of language education but also benefit pedagogical practices that support learners in their pursuit of English proficiency.

From the perspective of the Self-determination Theory (SDT) by Ryan and Deci [13], psychological needs can affect people’s behavior and well-being. Individuals have the psychological needs of autonomy and competence, according to SDT [13]. English proficiency can be viewed as a vehicle through which students satisfy their competence need. The mastery of a language is a demonstration of competence, providing individuals with the ability to navigate an interconnected world. Academic expectation stress from teachers, parents, or peers may compromise students’ autonomy. The proactive nature of adaptive coping aligns with SDT’s emphasis on autonomy. In actively addressing stressors and taking control of the coping process, students are fulfilling their need for autonomy. Academic resilience, characterized by a positive outlook, adaptability, and perseverance, resonates with SDT’s emphasis on psychological well-being. As a research gap in understanding the underlying mechanisms of latent variables that may directly or indirectly impact students’ EFL learning outcomes has been identified, this research introduced and tested a novel conceptual framework inspired by the theoretical perspective of SDT. Ultimately, this study aims to address the identified gap and contribute to a more comprehensive understanding of the factors influencing EFL learning outcomes.

## Theoretical underpinnings and hypothesis development

### Academic expectation stress and learning outcomes

The Transactional Model of Stress and Coping, proposed by Lazarus and Folkman [14], offers valuable insights into the relationships between academic expectation stress and learning outcomes. Lazarus and Folkman [14] assert that individuals evaluate stressors based on their cognitive appraisal, which comprises primary and secondary appraisals. Primary appraisal involves assessing the significance of the stressor, while secondary appraisal focuses on one’s ability to cope with it. In the context of academic expectation stress, students often appraise the stressor as significant and may question their ability to cope. According to Ang and Huan [15], this self-doubt can impair cognitive function, lessen their willingness to make efforts or engage, and ultimately have an effect on their learning outcomes. This self-doubt, combined with the emotional responses triggered by academic stress, can hinder concentration, information processing, and overall cognitive functioning [16]. This study therefore hypothesizes that academic expectation stress has a negative impact on EFL learners’ English proficiency. (Hypothesis 1)

### Academic resilience and learning outcomes

The relationships between academic resilience and learning outcomes can be explained with the Social Cognitive Theory [17]. The reciprocal interactions between people, the environment, and behaviors are highlighted in the Social Cognitive Theory. This theory contends that motivation, perseverance, and academic success are all greatly influenced by self-efficacy, or people’s perceptions of their capacity to complete particular tasks or goals.

Academic resilience, which involves students’ capacity to successfully navigate academic difficulties, setbacks, and stressors while maintaining a positive attitude and perseverance [18], is closely related to self-efficacy. According to Cassidy [18], students with high levels of academic resilience are more likely to have a strong sense of self-efficacy, which can affect their motivation to work hard, persevere in the face of challenges, and use effective learning strategies. Improved learning outcomes may result from this increased motivation and engagement.

Additionally, social modeling and observational learning are emphasized in the Social Cognitive Theory [19]. Students with high academic resilience may observe and model the behaviors of resilient peers, teachers, or mentors. By witnessing others’ successful efforts to overcome obstacles, students can develop self-efficacy beliefs and ultimately enhance their learning outcomes. Hence, this study hypothesizes that academic resilience positively predicts EFL learners’ English proficiency. (Hypothesis 2)

### Academic resilience as a mediator between academic expectation stress and academic performance

The Resilience Theory [20] offers valuable theoretical perspectives to explore how academic resilience may influence students’ ability to navigate stress and achieve optimal academic performance. The Resilience Theory [20] posits that individuals can adapt, thrive, and maintain positive functioning despite adversity. It emphasizes the dynamic process through which individuals harness their internal and external resources to cope with stress and overcome challenges. Resilience is not a fixed trait, but rather a malleable quality that can be fostered and developed [9].

Academic resilience may be crucial in mediating the connection between stress and academic performance in the context of learning. Academic resilience refers to a student’s capacity to overcome obstacles, stay motivated, and persevere [10]. Academic resilience can act as a protective factor and affect students’ academic performance when they are under high levels of academic expectation stress. Additionally, their resilience can mitigate the detrimental effects of stress on academic performance. Resilient students are more likely to see academic difficulties as growth opportunities rather than insurmountable obstacles. They view failures as temporary setbacks and maintain confidence in their abilities to overcome them [9]. This positive outlook and belief may ultimately lead to improved academic performance. Hence, this study hypothesizes that academic resilience mediates the relationship between academic expectation stress and EFL proficiency. (Hypothesis 3)

### Adaptive coping as a mediator between academic expectation stress and academic resilience

The Cognitive-behavioral Theory [21] may illuminate the mediating role of adaptive coping in the relationship between academic expectation stress and academic resilience. The Cognitive-behavioral Theory emphasizes the interplay between individuals’ thoughts, emotions, and behaviors [21]. This theory posits that individuals’ thoughts and interpretations of events significantly influence their emotional and behavioral responses. By recognizing and modifying maladaptive thoughts and behaviors, individuals can enhance their well-being and cope with stressors more effectively [21].

In the context of academic expectation stress and academic resilience, adaptive coping can be viewed through the lens of the Cognitive-behavioral Theory. Students who engage in adaptive coping strategies actively challenge and reframe negative thoughts and beliefs associated with academic expectation stress. They may replace self-defeating thoughts with more adaptive and empowering thoughts.

By adaptively challenging and modifying their thoughts, students can regulate their emotional responses to academic expectation stress, reducing anxiety, and increasing their resilience. These cognitive changes can lead to adaptive behaviors in the face of challenges, ultimately enhancing their academic resilience. Therefore, this study hypothesizes that adaptive coping mediates the relationship between academic expectation stress and academic resilience. (Hypothesis 4)

## This study

**Fig. 1:**
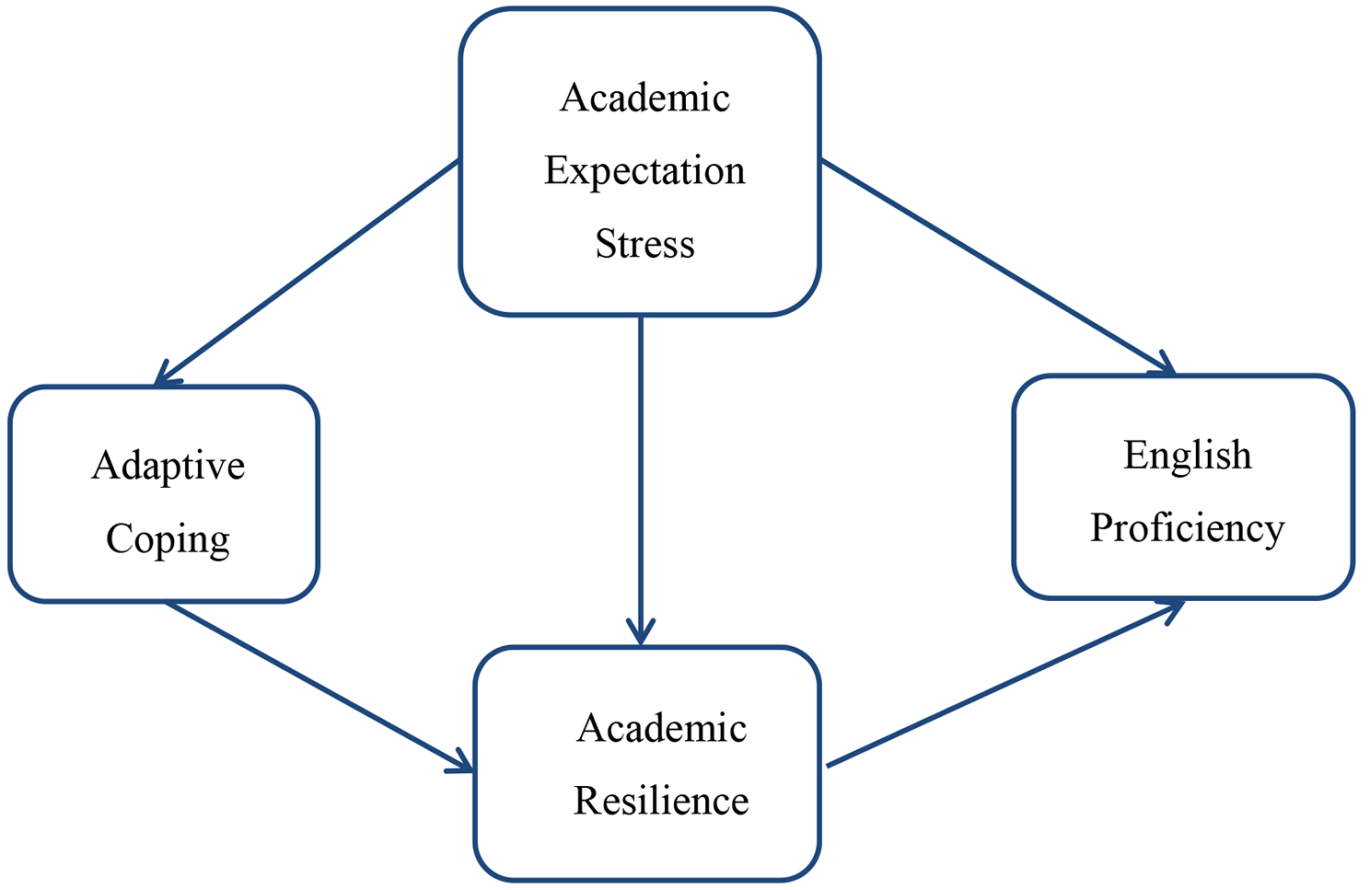
Conceptual framework

The four hypotheses are conceptualized as a research model as shown in Fig. 1. Four variables — academic expectation stress, adaptive coping, academic resilience, and perceived English proficiency — are examined in relation to one another in this study. Understanding how these factors interact and affect one another is the objective of the present study. This study aims to offer empirical evidence that quantifies the dynamics of these four variables by using a cross-sectional design in an EFL classroom setting at a university.

## Method

In order to examine the hypotheses and achieve the research objectives, this study employed a non-experimental research design to collect quantitative data. The study adopted a cross-sectional approach, engaging the involvement of college students who are currently enrolled in EFL courses. These students were invited to partake in a survey. The variables of interest were assessed through self-report measures, providing the participants with an opportunity to give their responses.

### Participants

The sample consisted of 395 young adults who are currently enrolled in EFL courses offered by a university in Taiwan, including 200 females (50.6%) and 195 males (49.4%). The participants had four age groups, 150 of them belonged to the category of 18 years old (38%), 199 students belonged to 19 years old (50.4%), 26 students belonged to 20 years old (6.6%), while 20 students belonged to the age category of 21 years old or above (5%). Notably, the majority of the participants belonged to the 18–19 age groups, accounting for approximately 90% of the total sample.

### Procedure

The study and the survey process were thoroughly explained to the EFL students before they took part in the survey. Consent forms were provided to inform the students about the goals of the current research project and to invite their participation. It was emphasized that the research data would be kept confidential for research purposes only and that their responses would not have an impact on their course scores. The students were given the freedom that they could end the survey at any time. All data collected were anonymous, and it took students approximately 20 min to complete the entire survey. The research qualifies as being exempt from ethical approval because it involves the use of non-sensitive, completely anonymous educational survey when the participants are not defined as “vulnerable” and participation does not induce undue psychological stress or anxiety. It is worth noting that this study followed the ethical guidelines established by the university.

### Measures

The Academic Expectation Stress Inventory (AESI) developed by Ang and Huan [15] consisting of nine items was used to measure the extent of academic stress resulting from personal expectations as well as those of parents and teachers. Each item in the scale is rated on a 5-point Likert scale, ranging from 1 (Never True) to 5 (Almost Always True). The total scores obtained from summing the item responses indicate the level of academic expectation stress, with higher scores indicating higher levels of stress. Ang and Huan [15] have established the AESI as a valid and reliable tool that has been successfully used among the adult population. This scale has been reported to possess good internal consistency (Cronbach’s α = 0.89) [15].

Adaptive coping strategies were assessed with the Adaptive Coping Scale (ACS) [3], which was derived from the active coping and planning subscales of the Coping Behavior Questionnaire (COPE) [3]. In the original study validating the COPE scale [3], the two subscales of Active Coping and Planning were found to converge into a single factor “adaptive coping”. Similar to the study by Thompson et al. [22], the author of the present study combined these two subscales to evaluate what was referred to as adaptive coping in this study. Students were asked to recall how they coped with academic expectation stress and rate each item on a four-point Likert scale, ranging from 1 (I usually don’t do this at all) to 4 (I usually do this a lot). A sample statement is “I take direct action to get around the problem.” Internal consistency for this scale has been reported to be α = 0.93 [22].

The Academic Resilience Scale (ARS) [9] was used to assess students’ capacity to proficiently handle setbacks, challenges, adversities, and pressures encountered within an academic environment. This scale has six items, and each of them is worded in a positive sense on a 5-point Likert scale, ranging from 1 (Never True) to 5 (Almost Always True). A sample item is “I’m good at bouncing back from a poor mark in my schoolwork.” This scale has been reported to possess good internal consistency (Cronbach’s α = 0.89) [9].

The participants’ perceived English proficiency was assessed using the Self-reported English Proficiency Scale (SEPS) [23], which consists of 12 items. This scale was developed based on previous studies conducted by Butler [24] and Chacon [25]. Participants rated each statement on a five-point Likert scale, ranging from 1 (strongly disagree) to 5 (strongly agree). A higher score indicates a higher level of perceived English proficiency. An example item from the scale is “In face-to-face interaction with an English speaker, I can participate in a conversation at a normal speed.” The SEPS has demonstrated good internal consistency, with a Cronbach’s α coefficient of 0.85 [23].

## Data analysis

This research involved an examination of demographic details, which are essential for testing the proposed relationships among study variables and validating theoretical propositions. To accomplish the objectives, SPSS v.27 was utilized.

To confirm that the collected data followed a normal distribution, values of skewness and kurtosis were examined and found to be approximately within the established criterions i.e., ± 1 or ± 2 [26, 27]. Furthermore, multicollinearity was assessed, and all variables in the model displayed VIF values below 3, indicating the absence of significant multicollinearity [26]. Consistent with Kock’s [28] recommendation, VIF values below 3.3 suggest no common method bias. Descriptive analysis, employing mean (M) and standard deviation (SD), was conducted to assess the central tendency and variability of the responses. Additionally, correlations among the study variables were examined to evaluate the nature and strength of the interrelationships. See Table 1 for details on above.

**Table 1:** Descriptive analysis

	M	SD	ACS	AESI	ARS	SEPS	Skewness	Kurtosis	VIF
ACS	2.91	0.53	1				-0.519	1.740	1.06
AESI	3.18	0.77	− 0.155^**^	1			-0.525	0.721	1.55
ARS	3.26	0.66	0.577^**^	− 0.231^**^	1		0.141	1.059	1.50
SEPS	3.10	0.64	0.381^**^	− 0.227^**^	0.342^**^	1	0.213	0.530	dv

Notes: ACS = Adaptive Coping Scale; AESI = Academic Expectation Stress Inventory; ARS = Academic Resilience Scale; SEPS = Self-reported English Proficiency Scale; M = mean; SD = standard deviation; VIF = variance inflation factor; *N* = 395, ** = *p* <.01, taken as dependent variable for VIF

### Assessment of measurement model

**Fig. 2:**
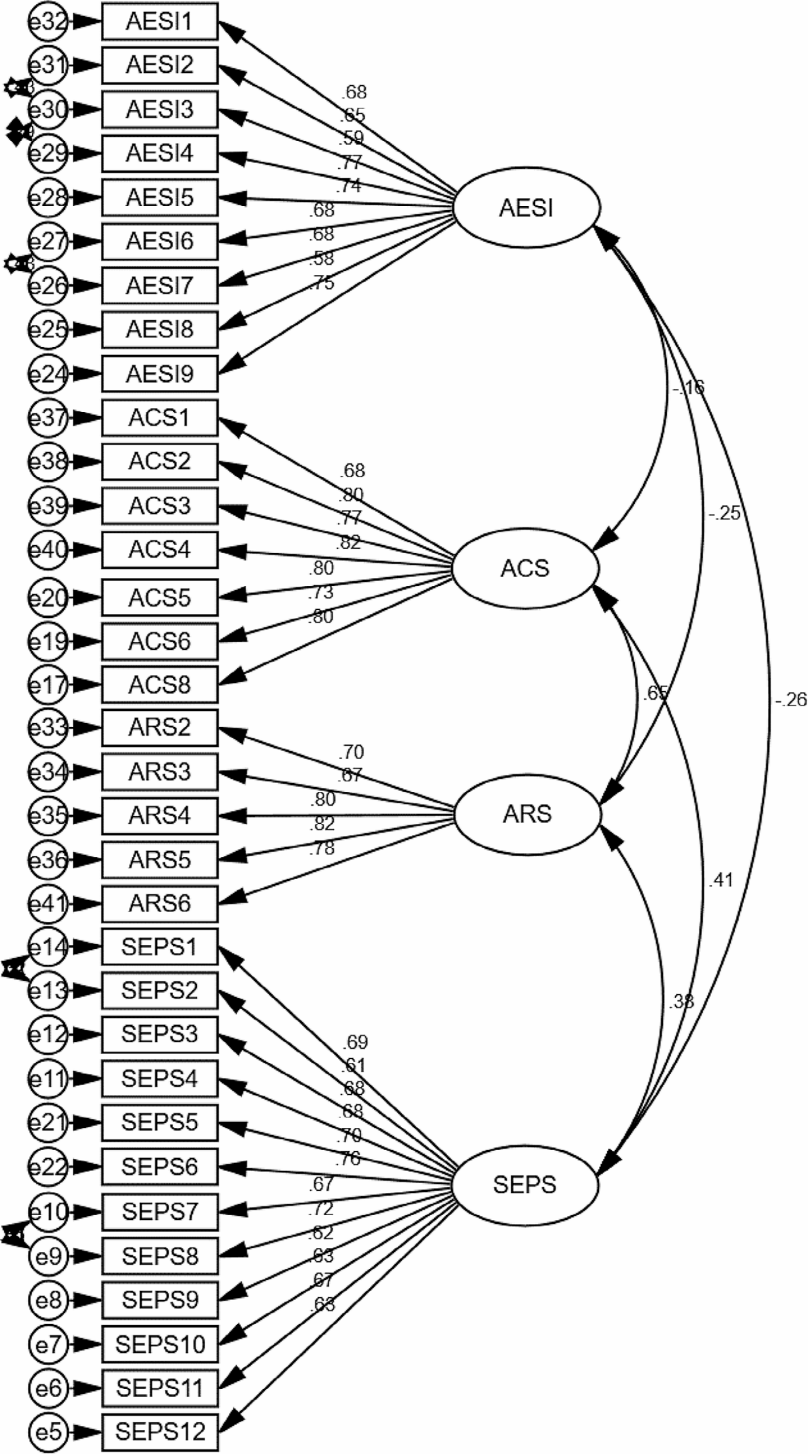
Measurement model diagram Notes: ACS = Adaptive Coping Scale; AESI = Academic Expectation Stress Inventory; ARS = Academic Resilience Scale; SEPS = Self-reported English Proficiency Scale; circles = error terms; double-headed arrows = measurement error correlations

Evaluation of the measurement model was done to assess the reliability and validity of the study scales. AMOS v.24 was utilized for this purpose. A four-factor confirmatory factor analysis (CFA) was conducted to examine the dimensionality and coherence of the ACS, AESI, ARS and SEPS factors [26]. In order to achieve the best possible fit between the data and the model, error terms were covariated based on modification index values exceeding 4 [26, 29], as depicted in Fig. 2. That four-factor model demonstrated a good fit, X^2^(984) / df (484) = 2.03 (< 3), RMR = 0.043 (< 0.08), TLI = 0.921 (> 0.90), CFI = 0.928 (> 0.90), and RMSEA = 0.051 (< 0.08). The acceptable criteria for these indices are provided in parentheses [26].

In addition to evaluating the model fitness indices, this study also ensured the reliability and validity of the measurement model through various additional procedures. Firstly, the factor loadings were examined, and items with factor loadings above 0.5 were kept (see Fig. 2). Cronbach’s alpha (CA) was also assessed, and all values exceeded 0.7, indicating satisfactory internal consistency. Composite reliability (CR) values were also above 0.7, further confirming the reliability of the measurement model. Convergent validity, assessed by the average variance extracted (AVE), exceeded 0.5 for all factors except for AESI and SEPS which had AVE values > 0.4. However, since their CR values were over 0.6, they were still considered acceptable [30].

**Table 2:** Reliability and validity analysis

Scale	CA	CR	AVE	HTMT				
				ACS	AESI	ARS	SEPS	
ACS	0.903	0.912	0.596	-				
AESI	0.891	0.886	0.465	0.172	-			
ARS	0.827	0.868	0.570	0.656	0.259	-		
SEPS	0.910	0.909	0.455	0.419	0.250	0.390	-	

Notes: ACS = Adaptive Coping Scale; AESI = Academic Expectation Stress Inventory; ARS = Academic Resilience Scale; SEPS = Self-reported English Proficiency Scale; CA = Cronbach’s alpha; CR = composite reliability; AVE = average variance extracted; HTMT = heterotrait–monotrait ratio

Additionally, the study evaluated discriminant validity through the Heterotrait-Monotrait (HTMT) ratio, ensuring that all ratios were below 0.85 [26]. This analysis reinforced the appropriateness of the measurement model, establishing a strong foundation for hypothesis testing, as presented in Table 2.

### Assessment of structural model

**Fig. 3:**
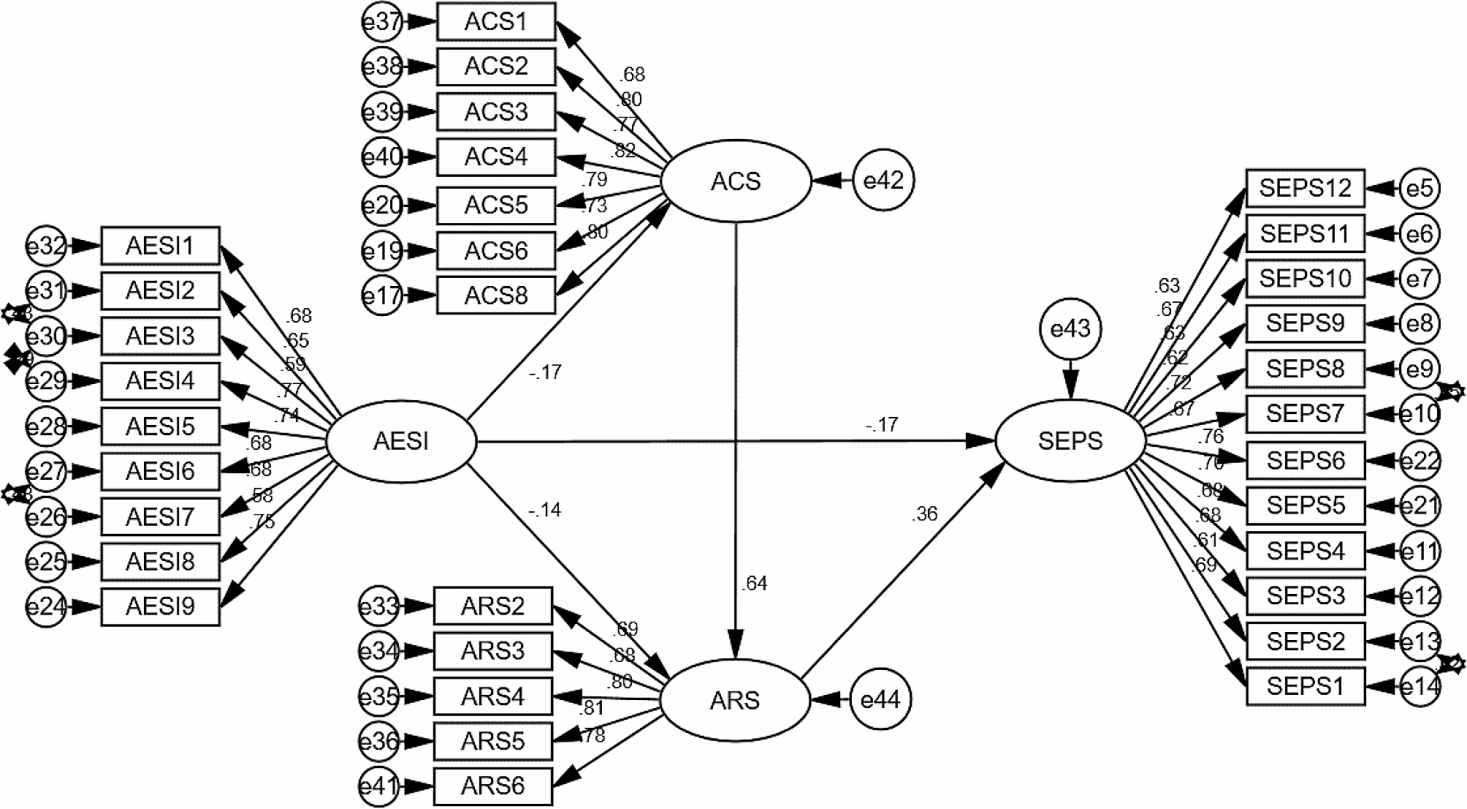
SEM path model Notes: ACS = Adaptive Coping Scale; AESI = Academic Expectation Stress Inventory; ARS = Academic Resilience Scale; SEPS = Self-reported English Proficiency Scale; circles = error terms; double-headed arrows = measurement error correlations

Evaluation of the structural model (depicted in Fig. 3) was done to evaluate its fitness and facilitate hypothesis testing. Based on the model fitness indices, the four-factor structural model demonstrated a good fit, X^2^ (1000) / df (485) = 2.06, RMR = 0.046, TLI = 0.919, CFI = 0.925, and RMSEA = 0.052 [26]. These results provided a strong basis for confidently testing the study’s hypotheses.

### Hypothesis testing

Hypothesis testing was conducted using structural equation modelling, employing 2000 samples of bias-corrected bootstrapping with 95% confidence intervals (CI) comprising lower bound (LB) and upper bound (UB). As illustrated in Table 3, the results revealed that AESI had a negative and significant impact on SEPS (B = − 0.167, *p* =.014, 95% CI [-0.282, − 0.036]), which confirmed that H1 was supported. It was concluded that academic expectation stress had a significant and negative impact on EFL learners’ English proficiency. ARS had a positive and significant impact on SEPS (B = 0.359, *p* =.001, 95% CI [0.227, 0.471]). Therefore, H2 was supported. It was concluded that academic resilience positively predicted EFL learners’ English proficiency. AESI had a negative and significant impact on SEPS through ARS (B = − 0.047, *p* =.011, 95% CI [-0.097, − 0.009]). Thus, mediation occurred. H3 was also supported. It was concluded that academic resilience mediated the relationship between academic expectation stress and English proficiency. AESI had a positive and significant impact on ARS through ACS (B = − 0.102, *p* =.013, 95% CI [-0.192, − 0.024]). Hence, again mediation occurred, so H4 was also supported. It was concluded that adaptive coping mediated the relationship between academic expectation stress and academic resilience.

**Table 3:** Hypotheses testing

Path	Estimate	LB	UB	*P*	Status
AESI $\rightarrow$ SEPS	− 0.167	− 0.282	− 0.036	0.014	H1: Supported
ARS $\rightarrow$ SEPS	0.359	0.227	0.471	0.001	H2: Supported
AESI $\rightarrow$ ARS $\rightarrow$ SEPS	− 0.047	− 0.097	− 0.009	0.011	H3: Supported
AESI $\rightarrow$ ACS $\rightarrow$ ARS	− 0.102	− 0.192	− 0.024	0.013	H4: Supported

Notes: ACS = Adaptive Coping Scale; AESI = Academic Expectation Stress Inventory; ARS = Academic Resilience Scale; SEPS = Self-reported English Proficiency Scale; LB = lower bound; UB = upper bound; *P* = *p* value

### Mediation analysis

While H3 was supported, the type of mediation was not determined based solely on the indirect effects. As indicated in Table 4, the direct effect of AESI on SEPS was found to be significant (B = − 0.158, *p* =.012, 95% CI [-0.275, − 0.037]), suggesting a case of partial mediation [31]. When ARS was introduced into the equation, the direct effect was decreased to B = − 0.047, meaning that ARS was able to lessen the negative impact of AESI on SEPS by 77.07% (indirect effect divided by total effect– 1). Sobel’s [32] test demonstrated that the indirect effect of AESI on SEPS via ARS was significant (z = -2.62, *p* =.009), providing additional support for H3. Similarly, for H4 it was a partial mediation as direct effect was significant (B = − 0.158, *p* =.012, 95% CI [-0.275, − 0.037]). ACS was able to lessen the negative impact of AESI on ARS by 60.77%. Sobel test also found to be significant (z = -2.84, *p* =.005), so it provided an extended support to H4.

**Table 4:** Mediation analysis

AESI $\rightarrow$ ARS $\rightarrow$ SEPS	Estimate	LB	UB	*P*	Status
Indirect Effect	− 0.047	− 0.097	− 0.009	0.011	Partial Mediation
Direct Effect	− 0.158	− 0.275	− 0.037	0.012	
Total Effect	− 0.205	− 0.318	− 0.085	0.001	
**AESI** → **ACS** → **ARS**					
Indirect Effect	− 0.102	− 0.192	− 0.024	0.013	Partial Mediation
Direct Effect	− 0.158	− 0.275	− 0.037	0.012	
Total Effect	− 0.260	− 0.423	− 0.091	0.003	

Notes: ACS = Adaptive Coping Scale; AESI = Academic Expectation Stress Inventory; ARS = Academic Resilience Scale; SEPS = Self-reported English Proficiency Scale; LB = lower bound; UB = upper bound; *P* = *p* value

## Discussion

The statistical findings reveal that academic expectation stress negatively predicts the perceived English proficiency of EFL learners, while academic resilience has a positive effect on perceived English proficiency. Concurrently, the relationship between academic expectation stress and perceived English proficiency is partially mediated by academic resilience. This study also reveals the mediating role of adaptive coping in the relationship between academic expectation stress and academic resilience. There has been a lack of research simultaneously exploring the interrelationships among these four variables in the existing literature. This study aims to fill this research gap by investigating their interconnectedness. While previous studies have primarily examined the individual links between these variables, this study stands out as one of the first to explore their simultaneous interplay. By doing so, it offers valuable quantitative evidence to deepen our understanding of these four variables within the research framework.

The statistical results revealed a significant negative effect of academic expectation stress on perceived English proficiency. There could be a couple of explanations for this finding. According to MacIntyre and Gregersen [33], high levels of academic stress may increase anxiety and have a negative impact, which can impede language learning. Academic expectation stress can negatively affect a learner’s capacity to learn and use English by impairing cognitive functioning, attentional focus, and information processing [15]. Stress can also make learners fear failure and create a tendency toward perfection [34]. Individuals who are burdened by intense academic expectations may find themselves compelled to meet or surpass these demands. Consequently, they may develop a predisposition to avoid errors rather than actively immerse themselves in purposeful language practice. The apprehension of failure can act as a hindrance to the acquisition of language, inhibiting learners from embracing challenges or experimenting with the language.

The analysis also unveiled a notable and beneficial impact of academic resilience on EFL learners’ perceived proficiency in the English language. This finding is similar to the results of previous studies [35, 36]. Academic resilience refers to the ability to rebound from setbacks and maintain motivated in the face of challenges [12]. It appears that EFL learners who possess a greater level of resilience are more likely to persist and exert effort in their language learning endeavors, even when confronted with difficulties. This sustained effort and motivation may contribute to an increase in language practice and involvement, which can ultimately lead to an improvement in the learners’ English proficiency. Moreover, resilient learners tend to possess a favorable perception of their academic capabilities [18], even when faced with obstacles. Having a higher level of self-efficacy may result in a heightened confidence in English language skills and a willingness to take on demanding language tasks. This positive mindset and self-belief may enhance the engagement of EFL learners in their language learning journey and positively impact the outcomes of English learning.

The relationship between academic expectation stress and English proficiency being mediated by academic resilience was also revealed in this study. Resilient learners may lessen the negative effects of academic expectation stress on EFL proficiency and advance positive English learning outcomes because they are more likely to perceive academic expectations stress as manageable and respond with proactive and effective strategies [20]. Additionally, as was already mentioned, students who are resilient tend to believe they are capable of overcoming obstacles and succeeding [18]. As learners with higher resilience are more likely to maintain confidence in their language learning abilities and persevere in the face of challenges, this positive self-belief can serve as a buffer against the detrimental effects of academic expectation stress.

The relationship between academic expectation stress and academic resilience was found to be mediated by adaptive coping, according to the statistical analysis. This finding can be explained with the Cognitive-Behavioral Theory [21]. Students may actively challenge and reframe unfavorable ideas and beliefs about academic expectation stress when they use adaptive coping strategies. In this process, self-defeating thoughts are swapped out for stronger, more empowering ones. Students can control their emotional responses to academic expectation stress by actively addressing and moderating their thoughts, which lowers anxiety and boosts resilience. When confronted with academic expectation stress, these cognitive changes and adaptive behaviors may ultimately improve their academic resilience.

### Implications

For EFL education, the finding that academic expectation stress has a significant negative impact on English proficiency has educational implications. This finding emphasizes the need to address academic expectation stress as a potential obstacle to English proficiency. Teachers might think about implementing student-centered approaches [37], which encourage a nurturing and supportive learning environment where students feel more empowered and less under pressure from their teachers, parents or themselves. EFL teachers can foster language learning by addressing academic expectation stress, as well as create an atmosphere conducive to language learning in order to better learners’ English proficiency.

The finding that academic resilience has a significant and positive impact on English proficiency emphasizes the importance of recognizing and fostering academic resilience in language learning. EFL instructors should think about incorporating practices and strategies that can improve students’ resilience, such as giving learners the chance to set goals and develop self-regulated learning strategies [38]. EFL instructors can help students overcome obstacles, stick with their language learning efforts, and ultimately raise their English proficiency by fostering academic resilience.

Pinpointing academic resilience as a mediator between the stress of academic expectations and English proficiency shows the significance of acknowledging and cultivating academic resilience as a crucial element in enhancing EFL proficiency. By fostering academic resilience, EFL educators can support students in effectively managing the pressures of academic expectations, ultimately resulting in enhanced EFL proficiency.

Similarly, the mediating role of adaptive coping in the relationship between academic expectation stress and academic resilience also carries implications for EFL education. To enhance learners’ academic resilience, EFL teachers can consider implementing interventions that help learners develop adaptive strategies such as active and planning approaches [3] to cope with academic expectation stress.

### Limitations

Like any research, it is important to recognize certain limitations of this study. Firstly, the reliance on self-report measures to assess the four variables introduces the possibility of response bias. Future investigations could benefit from incorporating objective measures and employing diverse research methods to gauge these variables. Moreover, the specific focus on EFL learners within the Taiwanese tertiary educational context may restrict the generalizability of the findings. Replicating the study in various learner populations and educational settings would offer additional perspectives on the interrelationship of these variables among EFL learners. Finally, for future researchers, there are still a few less explored psychological factors that could potentially influence EFL learning experience. One such factor is transpathy [39, 40]. It means the amount of emotional and sensory involvement of the teacher [39, 40]. Transpathy may affect students’ EFL learning experience. By incorporating transpathy into EFL research, future researchers can advance the field’s understanding of the complex dynamics of psychological factors at play in foreign language teaching and learning.

## Conclusion

Thus far, there has been a lack of comprehensive understanding regarding the interplay between academic expectation stress, adaptive coping, academic resilience, and perceived English proficiency among university EFL learners. This study aims to fill this research gap and expand the current knowledge base in this particular population. Moreover, the findings of this research have significant implications for EFL teachers who strive to enhance their students’ English proficiency. The author anticipates that these findings will contribute to the existing literature in the fields of education and applied linguistics by providing a thorough examination of these variables among college students. By gaining deeper insights into these variables, university EFL learners can improve their English learning experiences, while EFL instructors can offer better support to their students in enhancing their English proficiency.

## Data Availability

All data collected is available from the corresponding author upon reasonable request.
